# The opportunities and challenges of adopting ChatGPT in medical research

**DOI:** 10.3389/fmed.2023.1259640

**Published:** 2023-12-22

**Authors:** Abeer Alsadhan, Fahad Al-Anezi, Asmaa Almohanna, Norah Alnaim, Hayat Alzahrani, Reem Shinawi, Hoda AboAlsamh, Amal Bakhshwain, Maha Alenazy, Wejdan Arif, Seham Alyousef, Sami Alhamidi, Alya Alghamdi, Nour AlShrayfi, Nouf Bin Rubaian, Turki Alanzi, Alaa AlSahli, Rasha Alturki, Nawal Herzallah

**Affiliations:** ^1^Imam Abdulrahman Bin Faisal University, Dammam, Saudi Arabia; ^2^Princess Nourah Bint Abdulrahman University, Riyadh, Saudi Arabia; ^3^Northern Border University, Arar, Saudi Arabia; ^4^Eastern Health Cluster, Dammam, Saudi Arabia; ^5^Ministry of Health, Riyadh, Riyadh, Saudi Arabia; ^6^King Saud University, Riyadh, Riyadh, Saudi Arabia; ^7^Public Authority for Applied Education and Training, Kuwait City, Kuwait; ^8^King Saud bin Abdulaziz University for Health Sciences, Riyadh, Saudi Arabia; ^9^University College London, London, United Kingdom

**Keywords:** medical research, ChatGPT, healthcare, opportunities and application, challenges and successes

## Abstract

**Purpose:**

This study aims to investigate the opportunities and challenges of adopting ChatGPT in medical research.

**Methods:**

A qualitative approach with focus groups is adopted in this study. A total of 62 participants including academic researchers from different streams in medicine and eHealth, participated in this study.

**Results:**

A total of five themes with 16 sub-themes related to the opportunities; and a total of five themes with 12 sub-themes related to the challenges were identified. The major opportunities include improved data collection and analysis, improved communication and accessibility, and support for researchers in multiple streams of medical research. The major challenges identified were limitations of training data leading to bias, ethical issues, technical limitations, and limitations in data collection and analysis.

**Conclusion:**

Although ChatGPT can be used as a potential tool in medical research, there is a need for further evidence to generalize its impact on the different research activities.

## Introduction

Medical research seeks to further our understanding of human health and disease. It includes fundamental science, clinical research, epidemiology, and research on health services (1). The purpose of medical research is to increase our understanding of the underlying disease mechanisms, develop novel treatments and therapies, and improve healthcare delivery. A diverse collection of professionals, including scientists, physicians, nurses, pharmacists, and other healthcare professionals, conduct medical research (2). Typically, medical research involves a methodical and rigorous approach to data collection and analysis, frequently employing experimental and observational methods. This may involve testing novel drugs or therapies, investigating disease causes and risk factors, or assessing the efficacy of existing treatments and interventions (3). Medical research is essential for advancing our understanding of diseases and creating new treatments to enhance health outcomes. It has led to numerous significant medical discoveries, including the creation of antibiotics, vaccines, and innovative treatments for diseases such as cancer and HIV/AIDS.

Artificial Intelligence (AI) is playing an increasingly important role in medical research, and its applications are vast and varied. Some of the major areas in which AI is supporting medical research include: (a) Artificial intelligence can process and analyze vast quantities of medical data more rapidly and precisely than humans. This consists of medical images, patient records, and genetic information. AI algorithms can identify patterns and associations that may not be obvious to human researchers, thereby aiding in the identification of novel potential treatments and patients at risk for specific diseases (4). (b) AI can assist in accelerating drug discovery by predicting the safety and efficacy of novel drug candidates. Machine learning algorithms can analyze chemical compounds and predict their ability to interact with biological targets, thereby accelerating the drug discovery process and reducing the need for costly and time-intensive laboratory experiments (5). (c) AI can help tailor treatments to individual patients by analyzing genetic and other biological data to forecast the most effective therapies for a patient’s unique condition (6). (d) AI can analyze medical images such as X-rays, MRIs, and CT scans to aid in the diagnosis of diseases and the identification of abnormalities more swiftly and precisely (7). (e) By analyzing patient records and other data, AI can aid in the identification of prospective participants for clinical trials. This can aid in accelerating the recruitment process and ensuring that clinical trials are conducted with more representative patient populations (8).

AI is becoming an increasingly valuable tool for medical researchers, allowing them to analyze large amounts of data more precisely and efficiently, discover novel treatments, and enhance patient outcomes. Recent advancements in AI-based natural language processing and deep learning have led to the development of large language models such as ChatGPT. These models have been widely used for various applications such as language translation, text generation, and question-answering (9). ChatGPT operates as a sophisticated language model leveraging a transformer architecture, specifically GPT (Generative Pre-trained Transformer). Trained on a diverse corpus of text from the internet, it comprehends and generates human-like text responses based on the input it receives. Its capabilities encompass a broad spectrum of tasks, including language translation, text completion, question answering, summarization, and conversation. By understanding context, semantics, and patterns in the input text, ChatGPT generates coherent and contextually relevant responses, drawing from its vast knowledge base. It employs deep learning techniques to predict and generate text sequences, offering assistance, information, or engaging in conversation across various topics and domains, making it a versatile and powerful tool for natural language understanding and generation (10).

Studies (11, 12) have shown that ChatGPT can provide accurate and relevant answers to a wide range of questions, outperforming previous models in terms of accuracy and efficiency. Additionally, ChatGPT has demonstrated the ability to generate coherent and well-structured text, making it useful for applications such as content creation and summarization (13). Although ChatGPT has showed promise in several applications, issues with its possible biases and restrictions also exist. Studies have shown that language models like ChatGPT can reinforce pre-existing biases in the data they are trained on, producing biased results in their outputs. More study is required to solve ChatGPT’s shortcomings, which include those caused by the calibre and variety of the data used for training (14–16).

Researchers must be very mindful of any potential issues and drawbacks while using ChatGPT in the field of study, particularly when it comes to science and research in medicine. It is evident that ChatGPT may be used to produce manuscripts that could be viewed as plagiarised content because the authors did not create the content themselves. Another example of potentially dangerous use is when researchers utilise ChatGPT to generate a text that is extremely similar to a section or passages of text from a previously published work. A study’s outcomes could be manipulated or researchers could be misled by using ChatGPT to create language that is nearly comparable to previously published research (17–20). A recent study (21) examined whether or not human reviewers could tell the difference between actual scientific abstracts and abstracts produced by ChatGPT using AI. Blinded reviewers experienced difficulty telling the difference between abstracts produced by humans and those produced by AI. In fact, in 32% of the abstracts produced by the AI bot, ChatGPT was successful in deceiving blinded reviewers. The generated texts may lack context (as ChatGPT is trained using an extensive dataset of text but might not have sufficient information about a specific case), be inaccurate, biased (the training data may contain biases), and lack understanding of the nuances related to medical science(s) and language, among other risks and drawbacks, in addition to the plagiarism issue.

Recent studies (18, 22–27) have identified both opportunities and challenges of applying ChatGPT in medical research. The capabilities of ChatGPT emphasizes the increasing significance for scholarly publishing to employ robust AI author guidelines. Concerning copyright issues, the issue of attribution copyright infringement, and authorship, there are numerous ethical considerations when AI generates academic material. These issues are particularly significant because it is presently difficult for human readers and anti-plagiarism software to distinguish between AI-generated and human-written content. Various studies have already cited ChatGPT as an author (28), but it is debatable whether generative AI meets the criteria for authorship established by the international institutions of research and publication (22). Although, ChatGPT is available at free-of-cost, it could be temporary and the product may be monetized in the future (23). The commercial option might lead to disparities in scholarly publishing, as regions which are socio-economically backward may not be able to access, leading to the widening gap in knowledge dissemination and research. The usability and accessibility of ChatGPT could significantly increase scholarly output. ChatGPT could democratize the dissemination of knowledge because the chatbot can receive and generate copy in multiple languages, bypassing English-language prerequisites that can be an impediment to publication for speakers of other languages. Nevertheless, ChatGPT’s functionality has the potential to cause damage by generating misleading or inaccurate content, evoking concerns regarding scholarly misinformation (22).

In addition, other challenges include data privacy (in relation to patients’ data), limited availability of medical data (medical data is often limited in quantity and quality due to privacy concerns and the difficulty of collecting such data, which can limit the training for ChatGPT), quality, accuracy, and reliability in interpretation of results (25). However, ChatGPT could be the game changer in medical research in future, as with increasing data availability, its accuracy could improve; it can be integrated with electronic health records, leading to seamless exchange of information; and can support personalized medicine based on the personalized assessment of individual patients (26) in different areas of medicine (27). With its ability to understand and generate human language, ChatGPT can assist researchers in a variety of tasks such as literature review, data analysis and even the creation of new hypotheses. Due to its novel nature, its impact on education and research is not very well understood. Investigating the opportunities and challenges of adopting ChatGPT in medical research is important for ensuring that this technology is used to its full potential in advancing healthcare and improving patient outcomes. Therefore, this study aims to investigate the opportunities and challenges of adopting ChatGPT in medical research.

## Methods

### Study settings and sample

To investigate the opportunities and challenges of ChatGPT in medical research, a descriptive qualitative strategy employing focus group discussions (FGs) with academic researchers was considered. This technique facilitates the examination of issues and occurrences involving the perceptions and experiences of individuals (29). The inclusion criteria for the participants was a minimum of three publications in the last three years. To improve the diversity of the sample, participants from different streams including public health, medicine, eHealth, Health Information Systems were included. The focus group did not include participants other than medicine. As part of the recruiting phase for the FGs, an email invitation to participate in the study was sent to a convenient sample of academic researchers including students and professors, with an explicit inclusion criterion (those who are aware of ChatGPT). Those who consented to participate in FGs were invited to meetings at times that were convenient for them. Between December 2022 and April 2023, eight focus group discussions (FGs) were held at two universities with the participation of 62 academic researchers from a variety of specialties in medicine. To assure homogeneity and maximize the benefits of shared experiences, the number of participants per session ranged from seven to nine, depending on their practice backgrounds (30). Focus group participants were intentionally selected to ensure that a sufficient number of academic researchers from various streams in medicine participated in the study. The entire number of participants was determined using saturation. When the researchers reached a consensus that data categories had been established and new data had been sorted into these categories, the FGs were discontinued.

### Data collection

The FGs were created to collect information regarding the opportunities and challenges of ChatGPT in medical research. The researchers developed a comprehensive set of ten questions (Appendix A) to discuss the various effects or influences that ChatGPT in medical research. Follow-up questions such as “What do you mean?” and “Can you clarify, please?” were used to elicit additional information and stimulate more discourse. Because the sample involved participants from different countries, English (which is a commonly used medium of communication at Saudi Arabian universities) language medium was adopted for focus-group discussions. Every participant spoke English quite well, regardless of where they were from or what their mother tongue was. The interviewer who functioned as the FGs’ moderator ensured that all participants were involved by questioning about their perspectives on the study’s goals. This was done to ensure that all opinions were heard and to avoid the dominance of FGs by a few persons, which is a common concern with focus group processes. Despite this, the participants in this study had a lot in common. They were all academic researchers, and the interviewer was able to manage the FGs satisfactorily since the ChatGPT influence was constant in various ways. All participants were guaranteed anonymity and privacy, and their participation was completely voluntary. Voice recordings of the sessions were recorded in order to obtain complete information from the FGs. Every session lasted nearly an hour. Participants provided informed consent before to each FG session. The usage of pseudonyms and codes ensures that the data is reported in an anonymous manner. The ethical approval was obtained from the institutional review board of Imam Abdulrahman Bin Faisal University.

### Data analysis

The audio-recorded data was translated into text for computerized storage and management. Braun and Clarke’s approach was employed for theme analysis. The evaluation was guided by a method that entails examining and searching for noteworthy patterns and review topics in the data. This was followed by the development of early codes, the search for themes, the examination of themes, and the definition and naming of themes (31). Each topic group was given a name, and the coded data was organized and categorized using the MAXQDA 2022 program based on their similarity.

## Results

**Figure 1:**
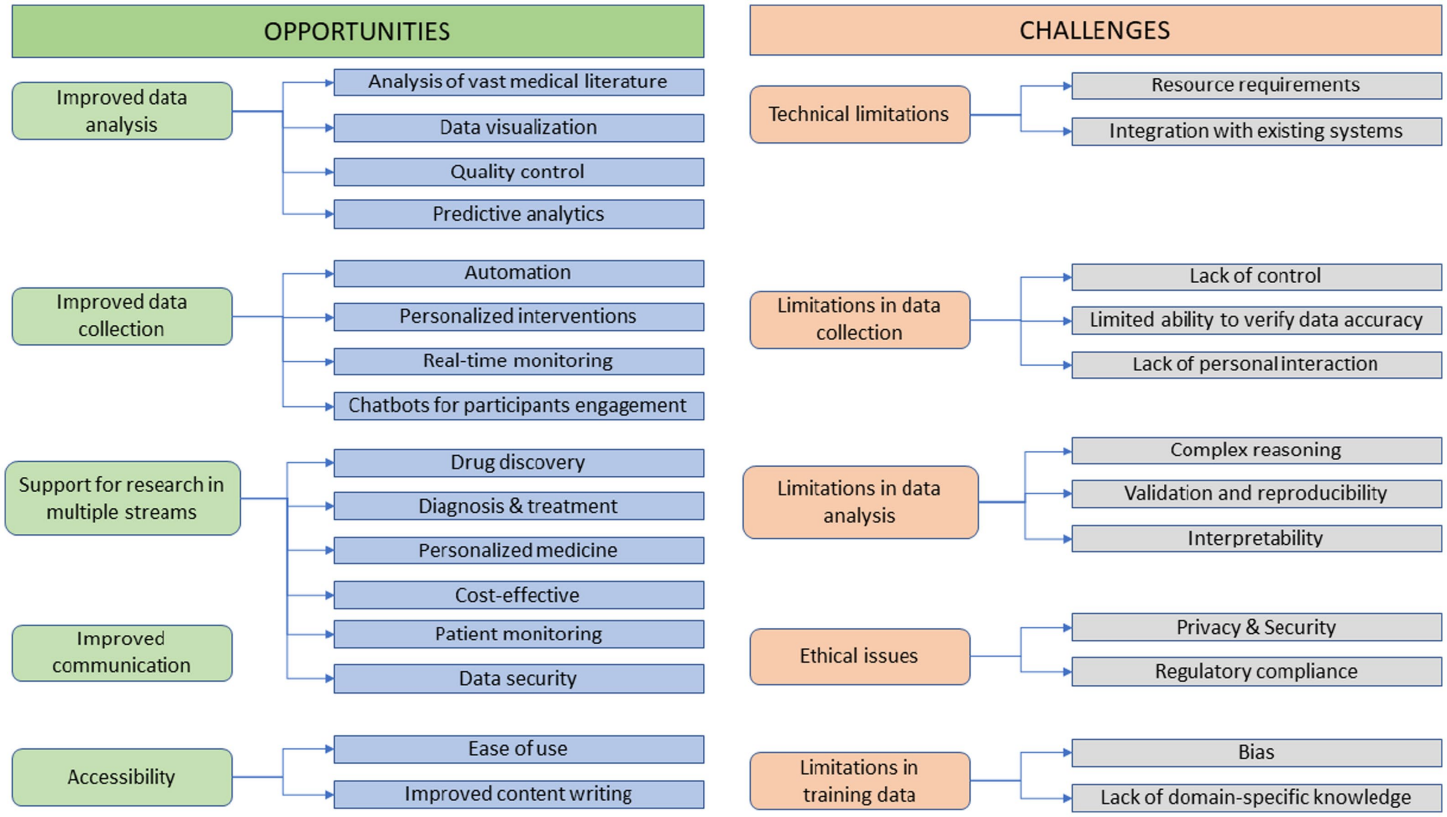
Opportunities and challenges of using ChatGPT in medical research.

Thematic analysis of FGs discussions resulted in ten main themes and 28 sub themes, as shown in Figure 1. These themes related to the opportunities of using ChatGPT in medical research include the following:

### Accessibility

More than 50% of the participants (41/62) opined that ChatGPT is an important tool that provide easy access in various research related works. These can be observed in usability and content writing.

#### Ease of Use

Few participants (19/62) observed that ChatGPT is easy to use compared to other platforms, as they can quickly identify the required information which can save time and resources. In this context, one of the participants stated that,

“Why should I google for any information and waste my time by browsing different links, when I can get the direct answer by simply typing a question on ChatGPT?”

#### Improved content writing

Support in content writing was observed by most of the participants (39/62). Most of them stated that they use ChatGPT in some areas of content writing.

“It is easy to formulate an idea to write some sections like introduction in the article by taking references from ChatGPT. Moreover, ChatGPT can also be used to identify the trending topics and new methods of critical analysis.”

#### Cost effective

As ChatGPT is now available for free, it is used by most of the researchers (41/62). One of the participants stated that.

“ChatGPT is very helpful. I had to pay to various platforms for accessing different publications related to my work, which proved to be costly for me. But using ChatGPT, I could get research-related information at free of cost.”

### Improved data collection

One of the important opportunities observed from the findings can be related to ChatGPT’s support in data collection. Its advantages are observed in different areas, which include:

#### Automation

Participants observed that ChatGPT model can be used for developing chatbots and other tools, which can automate the data collection process and reduce the burden on researchers and participants. In this context, one of the participants stated that.

“I think, ChatGPT based applications can be used for automating the data collection such as analysis of electronic health records which do not require human interventions.”

#### Personalized interventions

Few participants (23/62) observed that ChatGPT could be an effective tool for personalized based intervention studies like mental health disorders and diabetes. In this context, one of the participants stated that.

“ChatGPT may also be used with chatbots aimed at collecting information from patients such as their moods, activities that are essential for treating mental disorders, and the data can also be used for research purpose.”

#### Real-time monitoring

Similar to the automated and personalized interventions, real-time monitoring is another opportunity highlighted by few participants (12/62).

“By integrating ChatGPT with eHealth devices, we may get real-time data, which is automatically collected from the smart devices like diabetes chip or BP monitors; or personally collected data like sleep patterns and timings, positive and negative thoughts…”

#### Chatbots for participants engagement

One of the interesting findings is that some participants (23/62) perceived that sing ChatGPT based chatbots could engage participants in data collection. In relation to engagement, one of the participants stated that.

“Sometimes it is difficult to collect data from participants involving children. ChatGPT integrated chatbots could help in engaging such participants who finds chatbot interaction as funny and engaging…”

### Improved data analysis

Majority of the participants observed different uses of ChatGPT in data analysis related works. These include the following.

#### Analysis of vast medical literature

Most of the participants (57/62) observed that analyzing literature is a complex task, and they may miss-out critical information. In this context ChatGPT could be very helpful, as one of the participants stated.

“ChatGPT can analyze large amounts of medical literature and extract relevant information, such as specific symptoms, treatments, and outcomes. This can save researchers a significant amount of time and effort in conducting literature reviews.”

#### Data visualization

Few participants (11/62) observed that ChatGPT model along with other applications can be used to analyze complex data and generate charts or maps such as heat waves. In this context, one of the participants stated that.

“By integrating ChatGPT with data analysis tools, complex data analysis can be simplified. For instance, developing graphical designs based on large computational data like cell functions or cancer development, ChatGPT can help researchers to identify patterns and trends in their data. This can make it easier to communicate research findings and insights to stakeholders.”

#### Quality control

Few participants (17/62) opined that ChatGPT can be used to develop automated quality control processes to ensure data accuracy and completeness; this can help to improve the reliability and validity of research findings.

#### Predictive analytics

Almost 50% of the participants (30/62) observed that ChatGPT can analyze large datasets to identify patterns and trends, which can be used to develop predictive models for disease outbreaks like Covid-19, Ebola; patient outcomes; and other important factors.

### Improved communication: NLP/multiple languages

Most of the participants (48/62) observed that ChatGPT’s ability to understand and process natural language can help to improve communication between patients, researchers, and healthcare providers. This can lead to more effective treatment plans and better health outcomes for patients based on research findings. In this context, one of the participants stated that.

“ChatGPT can understand and process multiple languages, which can be useful in conducting cross-cultural research studies. This can help researchers to better understand cultural factors that may impact study outcomes and develop health interventions that are culturally sensitive and appropriate.”

### Support for research in multiple streams

Most of the participants observed the use of ChatGPT in different streams of medical research, which include the following.

#### Drug discovery

Few participants (21/62) observed that ChatGPT can assist in the identification of new drug targets and the prediction of potential side effects of drugs; which can help in accelerating the drug discovery process and lead to the development of more effective and safe drugs. Accordingly, one of the participants stated that.

“ChatGPT can be used in various areas of drug discovery like computing the compound multiplicity, generating inputs for gaussian and other software, identifying and validating the output and new targets.”

#### Diagnosis and treatment

Most of the participants (49/62) observed that ChatGPT could be an effective tool in disease diagnosis and developing personalized treatment plans, based on the input data. For instance, one of the participants stated.

“ChatGPT can assist in the analysis of medical data and patient records, which can help to identify patterns and insights that may be missed by human researchers.”

Therefore, ChatGPT can help in avoiding human errors in the disease diagnosis and treatment related research studies.

#### Personalized medicine

As mentioned above, few participants (9/62) observed that ChatGPT can be used to analyze individual conditions, and suggest personalized medicine accordingly. For instance, one of the participants stated that.

“ChatGPT can be used to analyze genomic data and identify specific mutations or genetic factors that may contribute to a patient’s condition. This information can be used to develop personalized treatment plans tailored to the individual patient.”

#### Patient monitoring

Few participants (15/62) observed the use of ChatGPT in monitoring patients with critical illness who require real-time monitoring. One of the participants in this context stated that.

“By integrating with monitoring applications, ChatGPT can be used to analyze genomic data and identify specific mutations or genetic factors that may contribute to a patient’s condition.”

This kind of information can be used to develop personalized treatment plans tailored to the individual patient.

#### Data security

Few participants stated that ChatGPT can also ensure data security if its integrated with other applications. In this context, one of the participants stated that.

“ChatGPT can help to ensure the privacy and security of patient data by encrypting sensitive information and restricting access to authorized personnel.”

This approach can help to protect patient confidentiality and comply with privacy regulations.

While the above presented results highlight the opportunities, the themes related to the challenges of using ChatGPT are presented below:

### Limitations in training data

One of the major limitations of ChatGPT as identified from the findings is limited training data. This was highlighted in different contexts, which include the following.

#### Bias

As ChatGPT relies on a large amount of training data to develop its language processing capabilities, it could lead to misinterpretations if the data is limited or incomplete or biased, as observed by many participants (32/62). This can be inferred from the following statements.

“In some medical research areas, there may be limited or incomplete training data available, which can impact the accuracy of ChatGPT’s language processing.”

“The training data used to develop ChatGPT may contain bias, which can lead to biased outputs. For example, if the training data primarily includes data from one population group, ChatGPT may be less accurate in processing data from other population groups.”

#### Lack of domain specific knowledge

It was observed that, as the ChatGPT is trained on large corpus data, it may not have specific knowledge related to a particular research domain (34/62). For instance, one of the participants stated that.

“In medical research, ChatGPT may not have specialized knowledge of anatomy, physiology, or pharmacology. This can limit its ability to process and analyze medical data accurately.”

### Limitations in data collection

Three limitations were identified from the analysis in relation to the use of ChatGPT for data collection, which include the following.

#### Lack of control

Few participants (27/62) observed that an integrated automated system with ChatGPT cannot collect data in a controlled and structured manner, which may lead to inconsistent or incomplete data that may be difficult to analyze or interpret.

#### Limited ability to verify data accuracy

Few participants observed that ChatGPT may not be able to verify the accuracy of the data collected in this context, one of the participants stated.

“In research studies where data accuracy is critical, ChatGPT’s inaccurate validation of data could seriously affect the analysis.”

#### Lack of personal interaction

Majority of the participants (42/62) observed the issue of personal interaction, especially in studies which involve human interaction like interviews. In this context, one of the participants stated.

“Automated Chatbots may not provide same levels of interaction that a human can with another person. It is important to understand emotions and many other psychological aspects in conducting interpretative studies, where ChatGPT could be ineffective.”

### Limitations in data analysis

In similar to data collection, there are challenges identified in relation to data analysis, which include the following.

#### Limitations in complex reasoning

Majority of the participants (39/62) observed that as ChatGPT is designed to generate text based on patterns in training data and may not be able to handle complex reasoning or logical inferences. This can be a limitation in research studies that require more advanced reasoning and analysis. In this context, one of the participants stated.

“In few areas, there is a need for complex analysis where we need to consider several influencing factors on a phenomenon, that require complex thinking and analysis, which could not be possible for ChatGPT.”

#### Validation and reproducibility

Researchers need to ensure that their results are reliable and reproducible, which can be challenging when using machine learning algorithms like ChatGPT, as observed by few participants (18/62). Issues like reliability, accuracy, validation were highlighted by the participants.

#### Interpretability

Very few participants (8/62) observed that it may be difficult to interpret or explain the output generated by ChatGPT in some cases, especially for non-experts. For instance, one of the participants stated.

“I use ChatGPT quite frequently, and I came across many situations, where its interpretations were unclear or wrong, and this sometimes confused me. As a result, I had to spend extra time in making it right, according to the phenomenon being investigated.”

### Ethical issues

Ethical issues are one of the important challenges highlighted by majority of the participants (59/62). These include the following:

#### Privacy and security

Most of the participants observed that medical data is highly sensitive, and researchers need to take measures to ensure that patient privacy is protected. As ChatGPT is an AI-based application, there are no clear strategies available to ensure privacy and security of the data. In this context, one of the participants stated.

“Ethical issues are an important challenge even for the existing researchers, who often make errors in the data collection and analysis process. For an application like ChatGPT, if integrated with other applications, there is a chance that privacy may be compromised in the world, where cybercrime is on the rise.”

#### Regulatory compliance

Most of the participants agreed that medical research is subject to strict regulatory requirements, and researchers need to ensure that they are complying with all relevant regulations and guidelines when using ChatGPT, which can be a complex and time-consuming process that requires careful planning and coordination.

“I am not aware of any regulations or standards that need to be followed while using ChatGPT in my research.”

### Technical limitations

Although ChatGPT was identified to be an effective AI tool, there are some technical challenges as observed by the participants, which include:

#### Resource requirements

Most of the participants (53/62) agreed that developing and deploying ChatGPT for medical research can require significant resources, including time, expertise, and funding, which can affect researcher’s ability to conduct research.

“ChatGPT is free for now, but it may be monetized soon, and it is being integrated with many applications of Microsoft, which may require premium costs and additional resources to make it customized to the institutional/researcher’s requirements. This can be challenging for researchers as it requires additional costs and resources.”

#### Integration with existing systems

Most of the participants agreed that integration of IT applications in medical research can be challenging as it requires expertise in both computer science and medical informatics; in addition to legal and regulatory aspects. In this context, one of the participants stated.

“Integrating ChatGPT with existing electronic health record (EHR) systems or other medical technologies can be challenging. There is a need for evidence-based research findings before assuming its usefulness.”

## Discussion

The purpose of this study is to analyze the opportunities and challenges of using ChatGPT in medical research. Accordingly, the data analysis resulted in 17 opportunities and 12 challenges as presented in the above section under the main themes. Focusing on the opportunities, it can be observed that accessibility is one of the core strengths of ChatGPT, which has led to its effective use in the areas of data collection, data analysis, improved communication, and support in multiple streams of medical research. Studies (23, 32–34) have observed the positive aspects of using ChatGPT in education and learning such as assistants for instructors and tutors for students; while few argued its negative effects such as ethical, copyright, transparency, and legal issues, the risk of bias, plagiarism, lack of originality, inaccurate content with risk of hallucination, limited knowledge, incorrect citations, cybersecurity issues (18, 35, 36). Recent studies focused on the data collection from a literature review perspective (37), which is secondary data collection. However, there is no empirical evidence identified by the researchers from the previous studies, where ChatGPT’s effectiveness is analyzed by using it as a data collection tool for primary data in research studies. However, there are studies that have analyzed the use of ChatGPT in data analysis, in analyzing the survey responses and social media data (38, 39), indicating a potential use of ChatGPT in data analysis. Another advantage of ChatGPT is that it can facilitate communication between researchers, analyze texts from different languages, which can support researchers from different backgrounds or cultures; and also due to its free availability, it can benefit disadvantaged researchers (22). ChatGPT’s potential has been also recognized in different streams of medical research such as drug discovery (40), diagnosis and treatment (41), personalized medicine (42), and patient monitoring, as identified from the findings in this study. These advantages of ChatGPT could help researchers in developing more quality research studies, and also benefit healthcare providers to take proactive measures to improve the healthcare operations with ChatGPT, and also prevent or manage disease outbreaks using predictive analytics. One of the key characteristics of AI is that it can be used for a variety of tasks in medical research without having to be retrained, which makes it more appealing compared to the traditional research process which requires vast amount of time and costs in training (43). For instance, AI plays a crucial role in efficiently analyzing large volumes of medical images, enabling the identification of illness characteristics that may go unnoticed by human observers (44). Realizing its potential, AI is being actively researched for its potential in medical research in the areas including medical image analysis (e.g., radiation, ultrasound, pathological image analysis), omics analysis (e.g., genome analysis), and natural language processing (e.g., EMR/EHR analysis) (45).

Despite its potential application in medical research, many challenges have been uncovered in the recent studies (46–50). Focusing on the challenges, ethical considerations was identified to be one of the key challenges that is affecting the use of ChatGPT in different research-related activities. Lack of regulatory compliances, issues with privacy and security were identified in recent studies (46–48). Researchers may need to develop secure data storage and transfer protocols to ensure that patient data is protected when using ChatGPT. Limitations in training data such as bias can affect the research findings, which could be a serious issue affecting the knowledge domain. Furthermore, dependency on training data for ChatGPT may limit its capability in domain specific knowledge, which can also affect the data collection and analysis, as identified in Liebrenz et al. (22). As observed from findings, in addition to lack of control over automated data collection using chatbots or other applications, and inability to verify data accuracy, lack of personal interaction is one of the major issues that can affect the quality of data collection and analysis. ChatGPT is an automated system and may not be able to provide the same level of personal interaction as human researchers. This can be a limitation in research studies that require personal interactions or interventions. However, it is important to understand not all studies require personal interaction, as a result of which ChatGPT could be useful in some studies. However, its reasoning capabilities, and interpretability aspects should be considered while using ChatGPT for data analysis. Resource requirements as identified by few participants as a challenge could be a short-term challenge, as many participants stated that ChatGPT is easy to use. However, its integration with other technical systems is an issue that need to be addressed (49). It was also observed that many online promotions were being carried out promoting ChatGPT, while its inability to produce meaningful content and other challenges associated with it are undermined (50), which needs to be addressed through empirical research on ChatGPT and its use in different streams including education and research.

Using ChatGPT for handling patient data and health information raises significant ethical considerations. Patient data is highly sensitive, and any mishandling, unauthorized access, or data breaches could lead to severe consequences for individuals, including privacy violations and compromised confidentiality. Moreover, ChatGPT’s responses might not always be entirely accurate or up-to-date in a rapidly evolving field like healthcare, potentially leading to misinformation or incorrect guidance. Therefore, employing ChatGPT for patient data and health information requires stringent safeguards, including robust data encryption, compliance with healthcare regulations like HIPAA, transparent communication about AI limitations, and constant validation of the information provided to ensure accuracy and reliability while prioritizing patient privacy and consent. Ethical oversight and continuous scrutiny are essential to mitigate potential risks and uphold the highest standards of ethical conduct in deploying AI in healthcare contexts. A recent study (51) has identified issues of ethics in medical research and recommended guidelines for reporting. In several AI extension guidelines in medical research, inclusion and exclusion criteria for patients and data types have to be precisely defined. Define the low-quality data assessment and processing method. The techniques should also state if humans selected which inputs were analyzed and which were eliminated. The standards advocate stating in the title, abstract, or both that the intervention uses AI for transparency. Second, techniques must indicate hardware, software, version(s), and internal thresholds studied. Third, the indication for use must be specified, including by whom (e.g., the user) and where along the clinical process. For imaging investigations, input must include technical requirements including image quality, field of view, resolution, camera device, and model. The result should match the indication for use and be clearly integrated into the clinical treatment route (51).

Overall, it can be argued that ChatGPT has considerably positive influence on its use in medical research; however, the challenges associated with it needs to be addressed. Moreover, there is a need for evidence-based research in this area to generalize the impact of using ChatGPT in medical research. Theoretical implications of this study involving ChatGPT in medical research suggest a significant shift in how AI transforms traditional research methodologies. It underscores the potential for AI-driven language models, like ChatGPT, to revolutionize data collection, analysis, and communication within the medical research domain. This study accentuates the evolving role of AI in augmenting researchers’ capabilities by providing efficient access to vast amounts of medical information, facilitating personalized interventions, aiding in data analysis, and improving cross-cultural communication in healthcare. The theoretical implications extend to the reconfiguration of research paradigms, emphasizing the integration of AI technologies into the fabric of scientific inquiry, leading to novel methodologies and improved research outcomes.

Practically, the study reveals tangible implications for the medical research community. ChatGPT’s utilization emerges as a practical solution to streamline various research processes, including content writing, data collection, and analysis. It presents a cost-effective alternative for researchers seeking information and support without significant financial investment. The integration of ChatGPT into medical research practices has the potential to enhance data accuracy, automate processes, and personalize interventions, thereby advancing healthcare delivery. However, the study underscores the need for caution, highlighting ethical considerations, limitations in data quality, and the necessity for interpretability and validation of AI-generated outcomes. These practical implications emphasize the need for careful integration, rigorous validation, and continual improvement in utilizing AI, like ChatGPT, as an adjunct in medical research endeavors.

There are limitations in this study. Firstly, the study’s reliance on focus group discussions with academic researchers, predominantly from medicine-related streams, might limit the diversity of perspectives, potentially overlooking insights from other fields relevant to AI or medical research. The sample’s composition might restrict the breadth of experiences and opinions, influencing the generalizability of findings. Additionally, while focus groups offer qualitative depth, they might not capture the full spectrum of opinions or nuances that individual interviews or mixed-method approaches could provide. The study’s qualitative nature might also lack quantitative validation of findings, limiting the broader empirical assessment of ChatGPT’s impact in medical research. Furthermore, the study does not deeply explore specific cases or actual implementation scenarios, potentially constraining the depth of understanding about real-world applications and challenges encountered when employing ChatGPT in diverse medical research contexts. These limitations warrant caution in generalizing the findings and underscore the necessity for further comprehensive, multi-method studies encompassing varied perspectives and practical implementations to enhance the understanding of ChatGPT’s role in medical research.

## Conclusion

The purpose of this study was to investigate the opportunities and challenges of using ChatGPT in medical research, and the findings have indicated that there are opportunities slightly outweigh challenges. However, there are few serious challenges like ethical and regulatory aspects which requires immediate attention, as the use of ChatGPT is significantly increasing among the researchers. As there is limited evidence on the impact of ChatGPT in medical research, there is a need to increase the research studies on the implications of using ChatGPT in medical research, especially in different research activities including developing research ideas, literature reviews, data collection, and analysis.

## Data availability statement

The raw data supporting the conclusions of this article will be made available by the authors, without undue reservation.

## Ethics statement

The ethical approval was obtained from the institutional review board of Imam Abdulrahman Bin Faisal University. Participants provided informed consent before to each FG session.

## Author contributions

AbA: Writing – original draft. FA-A: Supervision, Writing – review & editing. AsA: Formal analysis, Writing – review & editing. NorA: Writing – original draft. HaA: Writing – review & editing. RS: Resources, Writing – original draft. HoA: Writing – original draft. AB: Validation, Writing – review & editing. MA: Resources, Writing – original draft. WA: Methodology, Writing – original draft. SeA: Visualization, Writing – review & editing. SaA: Writing – original draft. AlyA: Resources, Writing – original draft. NouA: Project administration, Writing – review & editing. NR: Writing – original draft. TA: Writing – review & editing. AlaA: Software, Writing – original draft. RA: Visualization, Writing – review & editing. NH: Resources, Writing – original draft.
